# Pap smears in the extreme South of Brazil: low coverage and exposure of the most vulnerable pregnant women

**DOI:** 10.1590/1980-549720230032

**Published:** 2023-07-10

**Authors:** Juraci Almeida Cesar, Anelise Medeiros Souto, Carlota de Fátima Lelis, Larissa Picanço Pinheiro, Rinelly Pazinato Dutra, Rodrigo Jacobi Terlan

**Affiliations:** I Universidade Federal do Rio Grande, Postgraduate Program in Public Health, School of Medicine – Rio Grande (RS), Brazil.; II Universidade Federal do Rio Grande, Hospital Universitário Dr. Miguel Riet Corrêa Jr. – Rio Grande (RS), Brazil.

**Keywords:** Papanicolaou test, Prenatal care, Uterine cervical neoplasms, Risk factors, Equity in access to health services, Teste de Papanicolaou, Assistência pré-natal, Neoplasias do colo do útero, Fatores de risco, Equidade no acesso aos serviços de saúde

## Abstract

**Objective:**

To estimate prevalence, assess trends and identify factors associated with non-performance of Pap smears among postpartum women residing in Rio Grande, Southern Brazil.

**Methods:**

Between 01/01 and 12/31 of 2007, 2010, 2013, 2016 and 2019, previously trained interviewers applied a single standardized questionnaire at the hospital to all postpartum women residing in this municipality. It was investigated from the planning of pregnancy to the immediate postpartum period. The outcome consisted of not performing a Pap smear in the last three years. The chi-square test was used to compare proportions and assess trends, and Poisson regression with robust variance adjustment in the multivariate analysis. The measure of effect was the prevalence ratio (PR).

**Results:**

Although 80% of the 12,415 study participants had performed at least six prenatal consultations, 43.0% (95%CI 42.1–43.9%) had not been screened in the period. This proportion ranged from 64.0% (62.1–65.8%) to 27.9% (26.1–29.6%). The adjusted analysis showed a higher PR for not performing Pap smears among younger puerperal women, living without a partner, with black skin color, lower schooling, and family income, who did not have paid work during pregnancy or planned pregnancy, who attended fewer prenatal consultations. smoked during pregnancy and were not being treated for any illness.

**Conclusion:**

Despite the improvement in coverage, the observed rate of non-performance of Pap smears is still high. Women most likely to have cervical cancer were those who had the highest PR for not having this test.

## RESUMO

**Objetivo:**

Estimar a prevalência, avaliar a tendência e identificar fatores associados à não realização de citopatológico de colo uterino (CP) entre puérperas em Rio Grande (RS).

**Métodos:**

Entre 1o de janeiro e 31 de dezembro de 2007, 2010, 2013, 2016 e 2019, entrevistadores previamente treinados aplicaram, ainda no hospital, questionário único e padronizado a todas as puérperas residentes neste município que tiveram filho nos hospitais locais. Investigou-se desde o planejamento da gravidez até o pós-parto imediato. O desfecho foi constituído pela não realização de CP nos últimos três anos. Utilizou-se teste χ² para comparar proporções e avaliar tendência e regressão de Poisson com ajuste da variância robusta na análise multivariável. A medida de efeito utilizada foi a razão de prevalências (RP).

**Resultados:**

Apesar de 80% das 12.415 participantes do estudo terem realizado 6+ consultas de pré-natal, 43,0% (intervalo de confiança de 95% — IC95% 42,1–43,9%) não realizaram CP no período. Essa proporção variou de 64,0% (62,1–65,8%) a 27,9% (26,1–29,6%). Após a análise ajustada, puérperas de menor idade, cor da pele preta, sem companheiro, de menor escolaridade e renda familiar, que não exerciam trabalho remunerado, não planejaram a gravidez, realizaram menor número de consultas de pré-natal, fumaram na gravidez e não fizeram tratamento para alguma doença mostraram RP significativamente maior à não realização de CP em relação às demais.

**Conclusão:**

Apesar de melhora na cobertura, a taxa observada de não realização de CP ainda é elevada. Mulheres mais propensas a ter câncer de colo uterino foram as que apresentaram as maiores RP à não realização desse exame.

## INTRODUCTION

Although it is a preventable disease, easily detected and with a good prognosis, cervical cancer is one of the most important causes of illness and death in low- and middle-income countries^1,2^. In 2020, there were 604,000 new cases and 304,000 deaths worldwide, with at least 80% of them occurring in these countries^3^. According to the Global Cancer Observatory of the International Agency for Research on Cancer of the World Health Organization, cervical cancer is the third most common type of cancer among women aged up to 45 years in 146 of 189 countries evaluated^4^.

Brazil contributed with 17,700 new cases and 9,200 deaths, which gives the country a rate of 6.3 deaths per 100,000 women^5^. These coefficients hide important inequalities, from 3.7 to 12.6 deaths/100,000 women in the Southeast and North regions, respectively. In Rio Grande do Sul, in 2020, there were 720 new cases and 387 deaths, which corresponds to 5.8 deaths for every 100,000 women^6^.

Human papillomavirus (HPV) is a necessary factor for the development of cervical cancer, particularly subtypes 16 and 18, which account for 70% of all cases^6^. In addition to it, several other risk factors contribute to HPV infection, with emphasis on early sexual initiation, multiple partners, prolonged use of oral contraceptives, poor personal hygiene, sexually transmitted diseases, especially HIV, older age, brown or black skin color, low socioeconomic status, high parity, alcohol and tobacco consumption, living in rural areas, immunosuppression, and genetic predisposition^7–10^.

Since 2007, every three years, regular surveys have been carried out in Rio Grande (RS) with the objective of knowing indicators related to assistance during pregnancy and childbirth. These assessments include all puerperal women living in rural and urban areas of the municipality, use the same methodology, work with primary data collected solely and exclusively for this purpose, and have a rate of respondents of at least 98%. Among the indicators is the Papanicolaou test, aiming at the early detection of cervical cancer. The Brazilian Ministry of Health recommends that this test be performed for all pregnant women aged 25 years old or older, at any time during pregnancy^11,12^. Before this age, it should be avoided due to the low incidence of the disease, lower screening efficiency, and higher risk of obstetric and neonatal morbidity^5,12–14^.

This article estimated the prevalence, evaluated the trend and identified factors associated with not performing pap smear cervical cytology among puerperal women residing in this municipality between 2007 and 2019.

## METHODS

The present study was conducted in Rio Grande (RS), a municipality located in the extreme south of Brazil, whose headquarters are 250 km from the border with Uruguay and 300 km from Porto Alegre, the capital. With 212,000 inhabitants, Rio Grande is the tenth most populous municipality in Rio Grande do Sul. Located on the coast of the Atlantic Ocean, it has the second busiest port in Southern Brazil, essentially focused on the export of agricultural products. Port activity and agribusiness form the basis of its economy. Between 2008 and 2013, it experienced a certain “economic boom” due to the assembly of oil platforms, an activity that ended in 2016.

The public health network consists of two hospitals, one of them fully public, four medical specialty outpatient clinics, and 36 basic health units (*unidades básicas de saúde* – UBS). The Municipal Human Development Index reaches 0.744, and, for every thousand live births, 12 die before completing the first year of life, a value higher than the state mean, which is just over 10 deaths per thousand live births^15,16^.

This article is part of the Perinatal Studies of Rio Grande started 13 years ago with the objective of monitoring prenatal care and delivery offered in the municipality. The first of them was held in 2007, then came those of 2010, 2013, 2016, and 2019.

To be included in these surveys, the puerperal women must reside in an urban or rural area of the municipality, have given birth between January 1^st^ and December 31^st^ of those years and their children must have reached at least 500 g at birth and/or 20 weeks of gestational age. By including all postpartum women in the municipality and by addressing them only once, the research is characterized as a cross-sectional census study.

Within 48 hours after delivery, these puerperal women were interviewed by previously trained interviewers through a single, standardized, and pre-coded questionnaire, divided into blocks. These blocks sought information about the period from pregnancy planning to the immediate postpartum period. Considering the subject addressed in this article, only variables from the blocks of interest will be listed below, namely: demographic (maternal age and skin color, marital status); socioeconomic (education and current employment situation, family income); use of health services (prenatal care, gestational age and start of consultations, number of consultations, laboratory tests and clinical examinations performed, and location of prenatal care); morbidity during the gestational period (hypertension, *diabetes mellitus*, and depression); and life habits and behavior (smoking and alcohol consumption before and during pregnancy). There was a set of questions with regard to cervical cytology. Initially, it was asked if the puerperal women had taken a test to prevent cancer in the uterus/cervix/Papanicolaou or Cervical Cytology (CC) during pregnancy. In case of a negative answer, the reason for not having done so was asked. Then, they were asked whether they had taken this test at any time in the past. For positive answers, they were asked how long ago this had happened. Postpartum women who had not been submitted to a CC test in the last 36 months, but who should have^5,12^, were considered as not having performed it. While most of the variables are self-explanatory, four of them lack definition:

1. Family income: amount received by all those residing in the household in the month immediately preceding the interview;
2. Smoking: consumption of at least one cigarette per day in the six months prior to the interview and during at least one of the trimesters of pregnancy;
3. Intake of any amount of alcohol at least once a week during pregnancy; and
4. Adequate prenatal care, that is, starting consultations in the first trimester of pregnancy, having six or more prenatal consultations and at least two tests for HIV, syphilis, and a qualitative urine test.

This information was obtained by asking the mother directly and complemented based on a copy of the contents of the Pregnant Woman Health Card.

The questionnaires were always applied by four trained interviewers for 40 hours in the month prior to the start of data collection and who participated in a pilot study. This application took place through daily visits to maternity and inpatient wards every day of the week, including weekends and holidays.

In the 2007, 2010, and 2013 surveys, a physical questionnaire was used. On that occasion, the forms were coded and revised by the interviewers themselves and delivered to the headquarters of Perinatal Studies, on the premises of the School of Medicine of *Universidade Federal do Rio Grande* (FURG). The open questions were then coded, revised and typed twice by different professionals and in the reverse order of the first. This step was performed using the EpiData 3.1^17^ software. Comparison of the databases and subsequent correction was performed using the free software Epi Info^18^.

In the 2016 and 2019 surveys, data were entered simultaneously during the interview using tablets and the Research Electronic Data Capture (REDCap)^19^ app. At the end of each working day, these questionnaires were downloaded to the central server and then revised. Data analysis was performed using the Stata 12.0^20^ statistical package. The methodology used in the Perinatal Studies of Rio Grande is described in greater detail in another publication^21^.

The outcome of this study consisted of the failure to perform a cytological examination of the uterine cervix (or cytopathological examination of the uterine cervix) in the 36 months prior to the moment of delivery among those who should have done so. This period is also referred to here as the “last three years”.

The χ² test was used to assess the linear trend, while the multivariate analysis was performed using Poisson regression with robust variance adjustment^22^ and following a previously established hierarchical model (Chart 1)^23^. The effect measure used was the prevalence ratio (PR), with its respective 95% confidence interval (95%CI). Adjusted analysis included all model variables with p-value ≤0.20. Wald tests for heterogeneity and linear trend were used for ordinal exposures^24^.

**Chart 1:** Hierarchical analysis model for not performing uterine cervix cytology among postpartum women. Rio Grande (RS), 2007–2019.

Determinants	Level	Type of variable	Characteristic
Distal	I	Demographics	Age, skin color, and whether they live with a partner
		Socioeconomic	Education, family income (quartiles), paid work during pregnancy, and whether the husband is employed
Intermediary	II	Use of health services	Place of prenatal care, trimester in which prenatal care began, number of consultations performed, and whether the pregnancy was planned
Proximal	III	Life habits	Smoking (before and during) and alcohol consumption during pregnancy
		Morbidity	High blood pressure, diabetes, and depression
Outcome		Non-performance of cytopathology of the uterine cervix among puerperal women	

Approximately 10% of the interviews were redone by telephone within two weeks of the initial interview. The Kappa index of agreement ranged from 0.61 (planned pregnancy) to 0.99 (type of delivery), remaining between 0.72 and 0.91 for almost all of the evaluated variables, which is considered quite satisfactory^25^.

All research protocols were approved by the Research Ethics Committee in the Health Area (*Comitê de Ética em Pesquisa na Área da Saúde* – CEPAS) of FURG, which is linked to the National Research Ethics Committee (*Comitê Nacional de Ética em Pesquisa* – CONEP), under the following numbers: survey 2007 (Opinion 05369/2006); 2010 (Opinion 06258/2009); 2013 (Opinion 02623/2012); 2016 (Opinion 0030–2015); and 2019 (Opinion 278/2018).

## RESULTS

In the five surveys carried out, 12,663 mothers were identified. Of these, 12,415 were successfully interviewed, which corresponds to a response rate of around 98%. According the medical records of these puerperal women who were not interviewed because they left the hospital before the recommended minimum time, it was found that they were very similar to those interviewed in terms of demographic, reproductive, and health insurance characteristics. Therefore, it is possible to suggest that such losses did not significantly affect the results presented here.

Table 1 shows that about a third of the participants were 30 years old or older, 70% were of white skin color, 84% lived with a partner, 40% had higher education than elementary school, 43% had paid jobs during their pregnancy, 87% of their partners were employed, practically 80% started prenatal care in the first trimester and had at least six consultations, 58% of them in the public service, with two thirds of them having planned the pregnancy; 18% smoked and 4% consumed alcohol during pregnancy and just over half (54%) were being treated for at least one disease. Finally, 43.0% (95%CI 42.1–43.9%) of all of them did not undergo cervical cytology in the previous three years, ranging from 64.0% (62.1–65.8%) in 2007 to 27.9% (26.1–29.6%) in 2013. The trend analysis proved to be highly significant for almost all of the evaluated categories (Table 1).

**Table 1:** Distribution of postpartum women according to some demographic, socioeconomic, reproductive characteristics, life habits, and prenatal care received. Rio Grande (RS), 2007–2019.

Characteristic		Perinatal inquiry (%)					Mean 2007–19 (%)	p-value (trend)
		2007	2010	2013	2016	2019		
Maternal age (years)							p<0.001	
	11 to 24	48.7	45.5	44.0	33.2	40.6	44.4	p<0.001
	25 to 29	24.3 27.0	25.9	23.9	23.8	23.1	24.2	p<0.001
	30 to 47		28.7	32.0	33.2	36.3	31.4	p<0.001
Skin color							p<0.001	
	White	69.8	69.6	66.0	67.2	76.4	69.6	p<0.001
	Brown	18.3	20.6	22.4	22.6	15.2	20.0	p<0.001
	Black	11.9	9.8	11.7	10.2	8.4	10.4	p<0.001
Living with a partner		82.6	83.2	85.7	83.7	85.2	p=0.013	p=0.013
							84.1	
Education (years)							p<0.001	
	0 to 8	9.4	10.3	15.4	23.6	21.6	16.1	p<0.001
	9 to 11	41.8	44.6	44.7	39.7	47.2	43.5	p<0.001
	12+	48.8	45.2	39.9	36.7	31.2	40.4	p<0.001
Family income (quartiles)							p<0.001	
	1^st^ (worst)	32.3	26.3	16.5	22.7	29.5	25.3	p<0.001
	2^nd^	22.7	26.6	26.3	25.3	26.4	25.3	p=0.008
	3^rd^	21.6	23.1	25.7	25.3	27.0	24.5	p<0.001
	4^th^ (best)	23.4	24.0	31.5	26.8	17.1	24.8	p<0.001
Performed paid work during pregnancy		38.4	42.9	43.7	45.7	42.5	p<0.00142.5	p<0.001
Whether the partner was employed/working		84.7	89.4	92.3	83.2	84.9	p<0.001	p<0.001
							87.0	
Type of sector they had prenatal care in							p<0.001	
	Private	38.9	42.0	47.9	43.1	36.4	41.9	p<0.001
	Public	61.1	58.0	52.1	56.9	64.6	58.1	
Trimester when prenatal started							p<0.001	
	First	73.5	78.1	78.4	79.5	81.5	78.2	p<0.001
	Second	24.0	19.4	19.9	18.7	16.3	19.7	p<0.001
	Third	2.5	2.4	1.7	1.8	2.2	2.1	p<0.001
Had adequate prenatal care*		20.9	47.4	63.0	47.9	75.7	50.6	p<0.001
Number of prenatal consultations performed							p<0.001	
	0–1	5.6	5.8	3.3	2.0	3.9	4.1	p<0.001
	2–5	21.9	17.8	13.1	13.4	10.3	15.3	p<0.001
	6–8	37.9	36.1	37.3	38.8	31.3	36.4	p<0.001
	9–11	21.5	27.8	31.9	34.3	34.9	30.1	p<0.001
	12+	13.2	12.5	14.4	11.4	19.6	14.1	p<0.001
Planned pregnancy		63.3	63.8	62.8	60.3	67.0	p<0.001	p<0.001
							63.3	
Smoked before and during pregnancy		23.0	20.8	18.5	12.7	12.3	p<0.001	p<0.001
							17.5	
Used to drink alcohol during pregnancy		3.8	4.7	9.4	1.7	1.5	p<0.001	p<0.001
							4.2	
Were being treated for an illness during pregnancy†		65.3	57.5	59.1	48.1	39.2	p<0.001	p<0.001
							54.1	
Did not undergo CC in the last three years		64.0	35.8	27.9	44.6	42.6	p<0.001	p<0.001
							43.0	
Total	(%)	20.3	19.0	21.1	21.3	18.3	100.0	
	(n)	2,523	2,355	2,619	2,648	2,270	12,415	

CC: cervical cytology.

* Initiated prenatal care in the first trimester of pregnancy, had six or more consultations and at least two tests for HIV, syphilis and a common urine test;

† High blood pressure, *diabetes mellitus* and/or depression.

Table 2 consists of the prevalence of the outcome by category and crude and adjusted analyses. The prevalence by category of non-performing CC ranged from 30.5% among those with 12 years or more of schooling to 92.5% for puerperal women who did not undergo prenatal care or attended a single consultation. Puerperal women under 25 years of age, brown or black, living without a partner, with less education (0–8 years), belonging to the worst income quartile, who did not work outside the home during pregnancy, with an unemployed husband, who had fewer consultations, who had prenatal care in the private sector, who did not plan the pregnancy, who smoked during pregnancy and who were not being treated for any disease had a significantly higher PR for not performing a cervical cytology in the last three years in relation to the others.

**Table 2:** Prevalence by category and gross and adjusted analysis for factors associated with not performing cervical cytology among puerperal women who had children in the city of Rio Grande/RS, between 2007–2019.

Characteristic		Failure to perform cervical cytology % (n)	Prevalence ratio (95%CI)	
			Gross	Adjusted
Maternal age (years)		p<0.001	p<0.001	p<0.001
	11 to 24	49.2 (2,711)	1.00	1.12 (1.08–1.17)
	25 to 29	39.8 (1,196)	1.34 (1.28–1.41)	1.12 (1.05–1.14)
	30 to 47	36.7 (1,429)	1.09 (1.02–1.15)	1.00
Skin color		p<0.001	p<0.001	p=0.002
	White	41.3 (3,572)	1.00	1.00
	Brown	45.5 (1,128)	1.10 (1.05–1.16)	1.04 (0.98–1.09)
	Black	49.1 (636)	1.19 (1.12–1.26)	1.10 (1.03–1.18)
Living with a partner		p<0.001	p<0.001	p<0.001
	Yes	40.8 (4,257)	1.00	1.00
	No	54.6 (1,079)	1.34 (1.28–1.40)	1.21 (1.14–1.28)
Education (years)		p<0.001	p<0.001	p<0.001
	0 to 8	49.3 (2,477)	1.62 (1.51–1.74)	1.34 (1.23–1.45)
	9 to 11	41.7 (2,250)	1.37 (1.27–1.47)	1.25 (1.15–1.35)
	12+	30.5 (609)	1.00	1.00
Family income (quartiles)		p<0.001	p<0.001	p=0.001
	1^st^ (worst)	53.8 (1,629)	1.47 (1.38–1.57)	1.16 (1.08–1.25)
	2^nd^	43.3 (1,316)	1.29 (1.21–1.38)	1.09 (1.01–1.17)
	3^rd^	39.3 (1,153)	1.17 (1.09–1.25)	1.07 (1.00–1.16)
	4^th^ (best)	34.6 (1,027)	1.00	1.00
Whether they performed paid work during pregnancy		p<0.001	p<0.001	p<0.001
	Yes	37.4 (1,969)	1.00	1.00
	No	47.2 (3,367)	1.26 (1.21–1.32)	1.10 (1.04–1.15)
Employed partner		p<0.001	p<0.001	p=0.010
	Yes	41.2 (4,268)	1.00	1.00
	No	50.7 (785)	1.23 (1.17–1.30)	1.08 (1.02–1.14)
Type of sector they had prenatal care in		p<0.001	p<0.001	p<0.001
	Private	38.6 (1,941)	1.00	1.00
	Public	42.9 (2,997)	0.90 (0.86–0.94)	1.19 (1.13–1.26)
Trimester when prenatal started		p<0.001	p<0.001	p=0.307
	First	38.1 (3,567)	1.00	1.00
	Second	50.9 (1,199)	1.33 (1.27–1.40)	1.04 (0.99–1.10)
	Third	58.1 (147)	1.52 (1.37–1.70)	1.05 (0.92–1.19)
Number of prenatal consultations performed		p<0.001	p<0.001	p<0.001
	0–1	92.5 (468)	3.14 (2.91–3.39)	1.17 (1.07–1.27)
	2–5	56.1 (1,068)	1.91 (1.76–2.07)	1.34 (1.23–1.46)
	6–8	43.2 (1,954)	1.47 (1.36–1.59)	1.63 (1.49–1.79)
	9–11	35.6 (1,331)	1.21 (1.11–1.32)	1.87 (1.56–2.24)
	12+	29.4 (515)	1.00	1.00
Planned pregnancy		p<0.001	p<0.001	p<0.001
	No	35.9 (1,636)	1.31 (1.25–1.37)	1.13 (1.07–1.19)
	Yes	47.1 (3,700)	1.00	1.00
Smoked before and during pregnancy		p<0.001	p<0.001	p<0.001
	No	40.7 (4,169)	1.00	1.00
	Yes	53.8 (1,167)	1.32 (1.26–1.38)	1.14 (1.07–1.20)
Used to drink alcohol during pregnancy		p<0.001	p<0.001	p= 0.727
	No	42.7 (5,070)	1.00	1.00
	Yes	50.0 (266)	1.17 (1.07–1.28)	0.98 (0.88–1.09)
Were being treated for an illness during pregnancy*		p<0.001	p<0.001	p<0.001
	No	45.3 (2,582)	1.10 (1.06 1.16)	1.09 (1.05-1.14)
	Yes	41.0 (2,754)	1.00	1.00
Total (%) (n)		100.0 (5,336)	n=12,415	

* High blood pressure, *diabetes mellitus* and/or depression.

## DISCUSSION

There was an increase in CC coverage in the municipality over these 13 years. This caused the rate of not performing CC to fall from 64.0% in 2007 to 42.6% in 2019. The high number of consultations performed and the low coverage for CC observed are also noteworthy. With the exception of age below 25 years, the highest PR for not undergoing CC occurred among those with the highest risk for cervical-uterine cancer.

Most studies dealing with coverage for cervical CC in Brazil are restricted to women aged 25 years old or older and not necessarily pregnant women^26,27^, which makes comparisons with the results of this study difficult. Nevertheless, the 43% found as a total mean of not performing CC in the period is at least double the rate observed in other studies^28,29^. This is a result of the difference in the age group and the worsening screening for this disease, which began in 2013 and reached its peak in 2020^26,30^.

Younger age has been identified as a risk factor for not performing CC^22,27,30^. This is due to the fact that the incidence of invasive cervical cancer is very low among women up to 24 years of age, the low efficiency of screening for this disease, the fact that the low-grade lesions identified present a high probability of regression, the possibility of increasing the occurrence of obstetric and neonatal morbidity in a future pregnancy, especially premature birth, and low birth weight and premature membrane rupture^5,11–14^. Despite this, nearly half of the women (49.2%) had been submitted to this exam, which confirms that, in Brazil, screening is opportunistic, that is, women undergo Pap smears when they seek health services for other reasons. This means that about a quarter of the tests were performed outside the age group and in intervals much shorter than the recommended three years^5^. In Rio Grande, the mean obtained for these 13 years was twice as high as that reported by the National Cancer Institute José Alencar Gomes da Silva (*Instituto Nacional de Câncer José Alencar Gomes da Silva* – INCA), which reveals an excess of referrals, with a greater potential for risk over benefit among underaged women.

In Rio Grande, as skin color darkens, the probability of not performing cervical cytology increases. This fact was also observed in other studies and has been denounced for at least two decades^27–31,32^. This inequality, very evident when it comes to care during pregnancy and childbirth, has repeated itself, seeming to have been consolidated as a structural practice^32,33^. Coping strategies ought to be created. Empowering mothers and training health professionals in the proper handling of this issue should be part of this initiative.

The presence of a partner has been shown to be a protective factor for maternal and child health. With regard to performing CC in Rio Grande, it was no different. The possibility of undergoing CC among mothers who live with a partner was significantly higher compared to the others. This was also identified in other localities^29,33^.

Family income and education are invariably associated with maternal and child health indicators. As a rule, the higher the indicator, the better. In this study, as well as in several others, as income decreases and schooling worsens, the prevalence ratio for not performing CC grows higher^27,29–31^. Also in this study, it was verified that having an employed partner, as well as having a paid job during the gestational period, showed an effect on the (non) performance of CC. This group of variables operates in the same direction, with each of them having an independent effect on this outcome (Table 2). Improving maternal schooling should be a priority for all governments at their most different levels of management, due to its enormous positive impact on various indicators of maternal and child health.

As for prenatal care, it was found that the later the consultations are started, the greater the PR for not performing CC. This is because the number of consultations performed is lower and, therefore, the possibility of offering the exam is lower. In this sense, a succession of missed opportunities is evident. Table 1 shows that at least 80% of the mothers, while pregnant, had six or more consultations. Considering that with two consultations it would be possible to carry out the examination, hand in results and, if necessary, initiate treatment and management, the number of missed intervention opportunities is remarkable. Therefore, it is suggested that prenatal care is not being fully used for the prevention and early diagnosis of cervical cancer in Rio Grande. Efforts should be made to encourage the initiation of prenatal care in the first trimester of pregnancy and to offer this test as early as possible.

Similar to other studies, pregnant women who received prenatal care in the public sector had a higher PR for not undergoing CC compared to those assisted in the private sector (private doctor and/or health insurance)^27,28,30^. Having a greater number of prenatal consultations increases the probability of pregnant women undergoing CC. Despite the high number of consultations performed, coverage for CC was low, suggesting the need for a checklist of conducts and procedures to be offered in each consultation.

Mothers who did not plan the pregnancy showed a higher PR for not having CC. No other published study was found that investigated this association. However, it seems legitimate to assume that someone who becomes pregnant unintentionally also starts the prenatal consultations later and this ends up making it difficult to carry out all the necessary clinical procedures and tests. Unplanned pregnancy can be an indicator of not having CC.

As if smoking was not enough to harm the health of those who practice it and those who are exposed to it, in this study, it also appears to be independently associated with not performing CC. Women who smoked during pregnancy were less likely to undergo this test (PR=1.14; 95%CI 1.07–1.20). A similar result was observed in the Brazilian National Study on Health in 2013. In this study, after adjusting for several confounding factors, non-smoking women had an odds ratio (OR95%) of 1.66 (1.43–1.92) for performing of CC^2,30^. This suggests that smoking and poor health care may have the same determinants.

Finally, those postpartum women who were not treated for any disease during the gestational period were more likely to not undergo CC. In other words, this statement is equivalent to saying that those who go to the health service to treat a health problem are more likely to undergo CC, a fact already observed in other studies^27,28,30,31^. However, this is not so evident when the consultation is a prenatal one. If that were the case, CC coverage would be much higher than what was observed, given the profusion of prenatal consultations in the municipality. For this reason, it has been stated that prenatal consultations are not decisive for performing CC^30^.

When interpreting these results, it is necessary to consider at least three limitations that may have affected the present study:

1. The performance (or not) of CC was based on mothers’ reports, without documentary evidence. Because it is a desirable behavior or almost an obligation, it is possible that they mention having done it without actually having done so, leading to overestimation in the performance of the test;
2. It is also possible that interviewees confused performing CC (material collection) with gynecological examination (bimanual inspection and palpation). The difference between the two procedures was not explained to them at the time of the interview; and
3. The data presented here include a population not covered in the population surveys, that of puerperal women aged less than 25 years. This should be kept in mind when comparing these data with those from screening in the general population.

Our objective was to screen CC in prenatal care.

Despite the reduction in the performance of CC in Brazil as a whole from 2013 onward, there was an improvement in the coverage of this test in Rio Grande compared to 2007. The high coverage among women who should not have been submitted to the test should be noted. This suggests that as important as increasing coverage among those aged 25 years old or older is to improve the focus on providing this service, preventing those aged under 25 years from being subjected to a procedure that may bring them more risk than benefits. It is also necessary to prioritize care for the most vulnerable, since almost all cases of cervical cancer come from this group. Finally, the need to improve the quality of consultations is evident, to individualize the action offered in order to deliver the greatest possible benefit to each patient and to completely eliminate the possibility of causing them harm. If this does not happen, Rio Grande will continue to be a favorable place not only for the occurrence of cervical cancer, but also for its late detection.
